# Sigmoid neovagina prolapse treated with Altemeier procedure: case report and systematic review of the literature

**DOI:** 10.1007/s00192-023-05603-4

**Published:** 2023-07-25

**Authors:** Kristina Drusany Starič, Rosario Emanuele Carlo Distefano, Gregor Norčič

**Affiliations:** 1 Department of Gynecology, Division of Gynecology and Obstetrics, University Medical Centre Ljubljana, Faculty of Medicine, University of Ljubljana, Ljubljana, Slovenia; 2 Ist. Patologia Ostetrica E Ginecologica, Department of General Surgery and Medical Surgical Specialities, University of Catania, Via Santa Sofia 78, 95100 Catania, Italy; 3 Department of Abdominal Surgery, Division of Surgery, University Medical Centre Ljubljana, Faculty of Medicine, University of Ljubljana, Ljubljana, Slovenia

**Keywords:** Neovagina prolapse, Vaginoplasty, Sigmoid neovagina, MRKH, Mayer–Rokitansky–Küster–Hauser syndrome

## Abstract

**Background:**

Bowel vaginoplasty is a surgical method for neovagina construction that, despite its advantages over other techniques, is still burdened by complications such as prolapse. The incidence of sigmoid neovagina prolapse (SNP) is difficult to determine, and there are no evidence-based recommendations for treatment. We present a case of SNP and a systematic review of previous cases.

**Case:**

A 73-year-old woman presented with stage III prolapse of her sigmoid neovagina constructed 51 years prior. Dynamic pelvic MRI revealed that the majority of the prolapse was due to the mucosa’s loss of support. Due to the presence of numerous pelvic adhesions, an alternative to the laparoscopic approach was evaluated by a multidisciplinary team which led to the patient being treated using a modification of Altemeier’s procedure.

**Systematic review:**

After PROSPERO Registration (CRD42023400677), a systematic search of Medline and Scopus was performed using specific search terms. Study metadata including patient demographics, prolapse measurements, reconstruction techniques, recurrence rates, and timing were extracted. Fourteen studies comprising 17 cases of SNP were included. Vaginal resection of the redundant sigmoid, comprising Altemeier’s procedure, was the most definitive surgery, but it was also associated with recurrences in three cases. Laparoscopic sacropexy was the second most definitive surgery with no recurrence reported.

**Conclusion:**

Our review shows that the recurrence after correction of sigmoid neovagina prolapses is higher than previously reported. Laparoscopy colposacropexy appeared to be the best approach, but it’s not always feasible. In these scenarios, a mucosal resection using the Altemeier’s procedure is the most effective surgery.

## Introduction

Patients presenting with a neovagina prolapse can be challenging for many reasons. First of all, it’s a rare condition and poorly studied. Most importantly, the term neovagina is very generic and it encompasses different surgical and non-surgical treatments that can be used to create a functional vagina, and many modifications of these treatments exist. Thus, each different method of reconstruction can lead to a different new anatomical setup that requires a tailored approach.

Furthermore, the prolapse can develop many years after the construction of the neovagina, and this could make it difficult to retrieve any valuable information regarding the first surgery.

Hereby we present our case report of a 73-year-old woman with a POP-Q stage 3 prolapse of a sigmoid neovagina that was created 51 years before, in which a modification of the Altemeier procedure was used to correct the prolapse. The case report was prepared following the CARE Guidelines [1].

In order to describe the different options available and their flaws, a systematic literature review of all previous cases of sigmoid neovagina prolapse was carried out.

## Case report

### Patient information

**Fig. 1:**
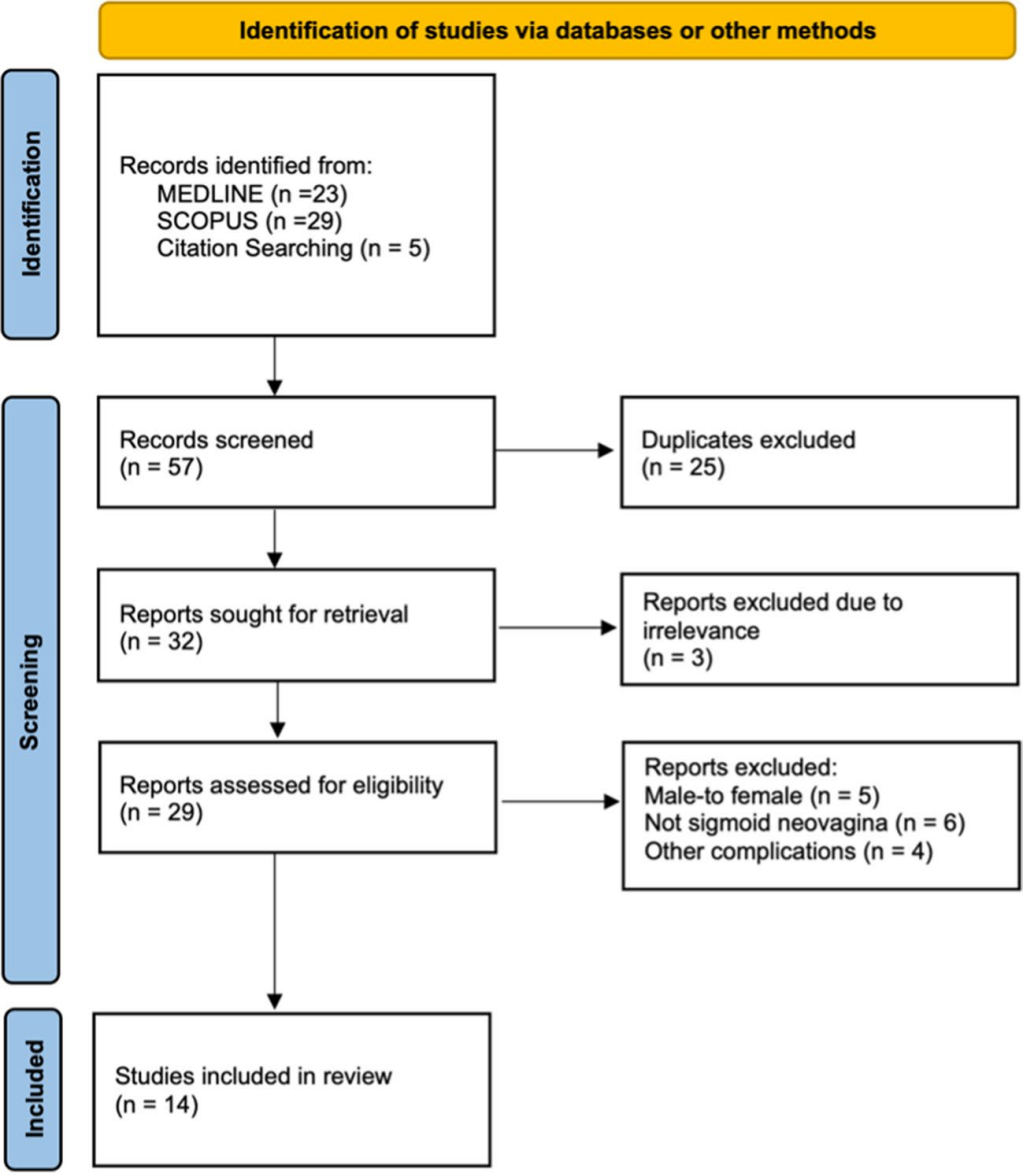
PRISMA flowchart

A 73-year-old, with Mayer–Rokitansky–Küster–Hauser (MRKH) syndrome (karyotype 46, XX) presented to our service complaining of a vaginal-bulge sensation, voiding difficulties and continuous vaginal discharge. She stopped sexual activity with her husband because of the physical and psychological discomfort the bulge was giving her. Because of her vaginal aplasia, she had undergone abdominal sigmoid vaginoplasty in 1969, at 22 years of age, which was uneventful and permitted her to have a normal sexual life till recently. She was a non-alcoholic and nonsmoker and apart from a history of hypertension and allergic asthma, she didn’t report any significant pathology (Fig. 1).

### Clinical findings

**Fig. 2:**
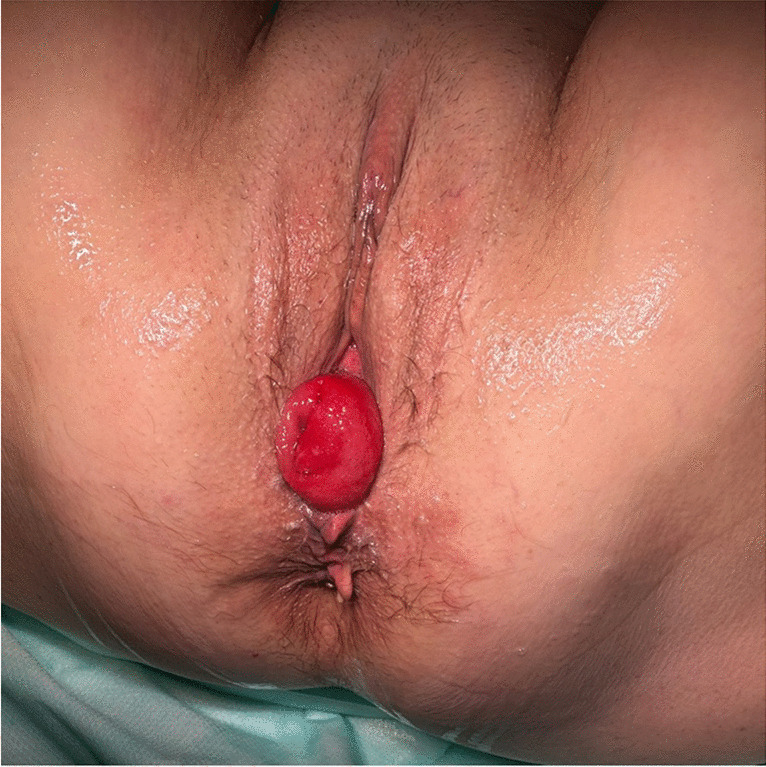
Neovagina prolapse

On her first visit, the gynaecological examination showed normal secondary sexual characteristics, her abdomen was palpable with no rebound tenderness, and her external genital appeared normal. After placing the patient in the dorsal lithotomy position, a full-thickness prolapse of the neovaginal mucosa was noticed on maximum Valsalva, which was a stage 3 prolapse according to the pelvic organ prolapse quantification system of the International Continence Society ([POP-Q]: Aa + 1, Ba + 5, C + 5 Ap + 3, Bp + 5, TVL 12) (Fig. 2).

### Diagnostic assessment and therapeutic interventions

Since the patient did not have any medical records with regard to the procedure that was carried out 51 years ago to create the neovagina, we decided to perform a diagnostic laparoscopy with the intent to perform colposacropexy if deemed feasible. When the pelvic cavity was examined, the course of the neovagina could not be clearly delineated due to significant adhesions. Several attempts were made in order to free the neovagina and expose its anterior and posterior walls. The rectovaginal space could not be dissected, and in fear of damaging the original sigmoid's vascular pedicle, we decided to cease the procedure.

**Fig. 3:**
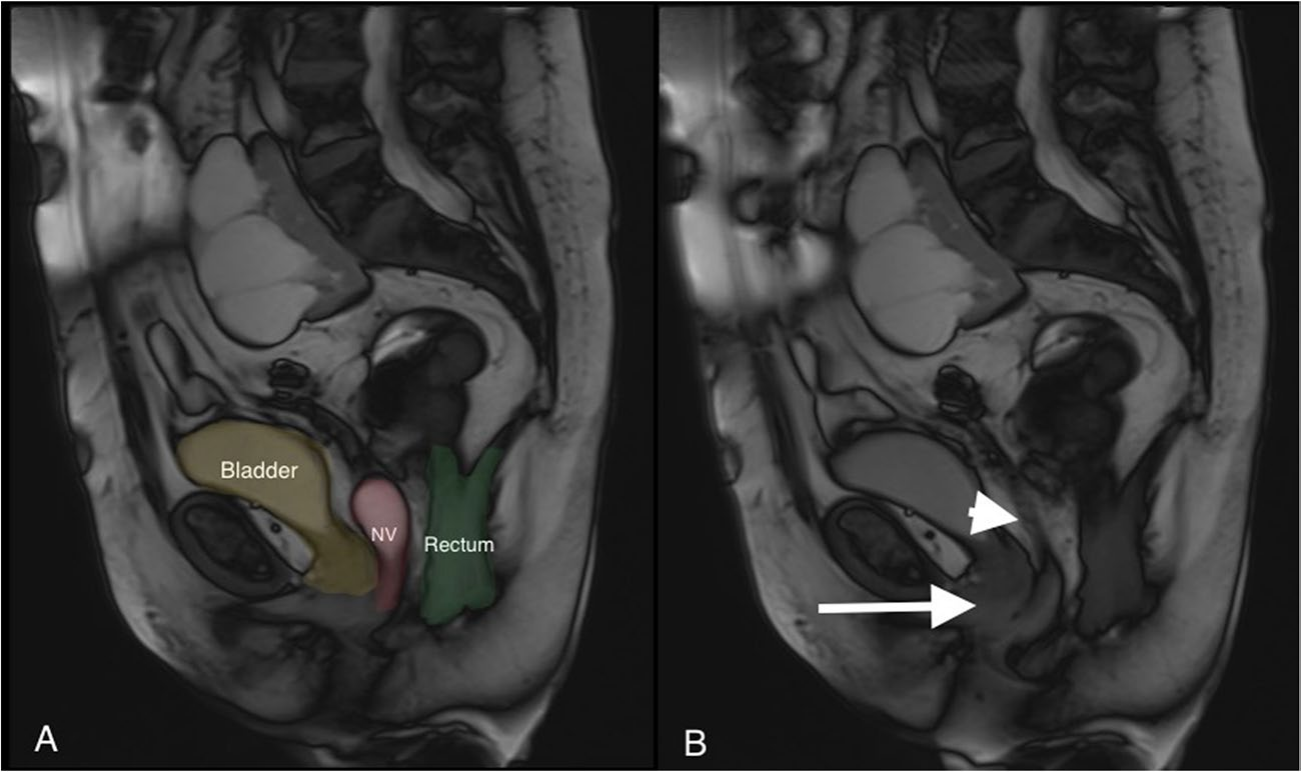
Dynamic pelvic MRI. **A** At rest, **B** at maximum Valsalva full-thickness prolapse of the mucosa of the neovagina (NV) with intussusception shown (*white arrow*) with the remaining neovagina staying in place (*head arrow*)

A pelvic CT scan and an MRI defecating proctography were ordered to better study the anatomy of the patient. The CT scan could not identify the origin of the neovagina's vasculature, but it pointed out the presence of an ectopic multicystic kidney that localized the level of L4-S1. The dynamic pelvic floor MRI showed that the pelvic wall muscles were thin and that the axis of the neovagina was oblique going from right to left and upward, probably because of the presence of an adipose tissue bulge on the dorsal-left wall of the vagina. On Valsalva manoeuvre, the MRI confirmed vaginal vault-only prolapse, with just a slight descent of the bladder and anorectal angle, and showed that the prolapse predominantly consisted of the adipose tissue associated with mesentery of the sigmoid neovagina (Fig. 3). Furthermore, intussusception of the neovaginal mucosa was noted.

Unfortunately, during our diagnostic workup, the patient was diagnosed and surgically treated for a tubulopapillary carcinoma of the esophagogastric junction (Siewert–Sten II–III), and our intervention was consequently postponed.

Approximately 2 years later she came back to our service. A multidisciplinary team meeting of gastrointestinal surgeons and urogynaecologist was held, and her situation was reevaluated. Due to difficulties encountered on our first attempt to release the neovagina from the dense adhesions, a decision was made to treat the patient by a vaginal approach, with the intention to perform a modification of the Altemeier’s procedure used for rectal prolapse.

We discussed our decision with the patient and informed consent was obtained. The patient's written consent was also obtained for the use of the photographs.

**Fig. 4:**
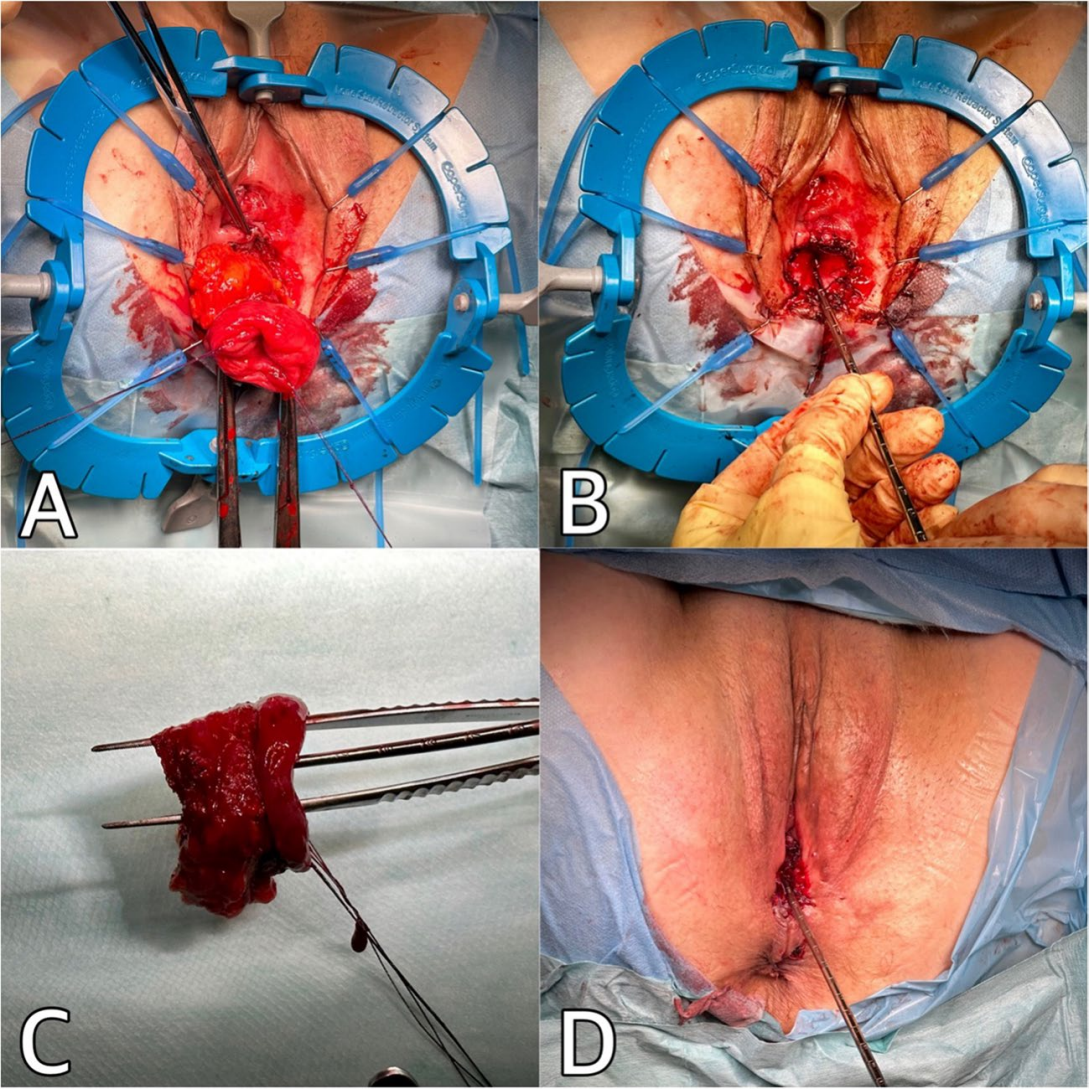
Altemeier procedure for sigmoid neovagina prolapse: **A** Dissection of the mesosigma. **B** TVL at the end of the procedure measuring 6 cm before removal of the retractor. **C** Resected sigmoid neovagina with hysterometer showing an approximated length of 3 cm. **D** Appearance of genitalia at the end of the procedure

The patient was placed in the lithotomy position and the Lone Star retractor was set (Fig. 4). Similar to the original Altemeier procedure for rectal prolapse, light traction was applied after transection of the sigmoid neovagina at the muco-cutaneous border. This facilitated the eversion of the entire mobile portion of the sigmoid, allowing for clear visualization of the extent of the prolapse. The proximal resection was subsequently performed at the level of the introitus, ensuring the establishment of a tension-free anastomosis. By adhering to this approach, the aim was to achieve a suitable balance between removal of the prolapsed segment and preservation of an adequate length of the neovagina. The mesosigma was then dissected with an advanced bipolar energy device. The resected neovagina, measuring approximately 5 cm, was sent for histopathological examination. Holding sutures were placed around the entire mucosa, followed by the suturing of the neovagina walls using Vicryl 3.0 sutures along the entire circumference. The depth of the remaining vagina was assessed at the conclusion of the procedure, and measured approximately 6–7 cm. The vaginal mucosa was vital and pink.

The postoperative course was uneventful, and the patient was discharged on the third postoperative day.

### Follow-up and outcomes

Histopathology of the specimen reported non-specific inflammatory changes in the mucosa but otherwise no other changes.

**Fig. 5:**
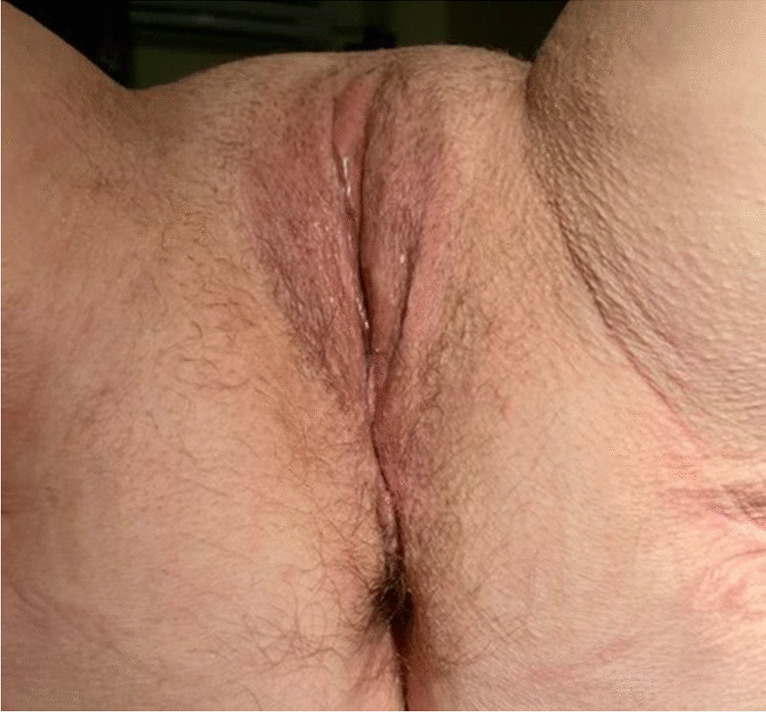
Photo taken at 6-month follow-up visit

At her six-month follow-up, the patient reported significant improvement in symptoms and overall quality; at the resumption of sexual activity she initially complained of mild dyspareunia. When examined, her POP-Q was Aa -3, Ba-3, C -5 Ap -3, Bp -3, TVL 6 (Fig. 5). Since the recurrence of prolapse is possible, we planned to continue our follow-up for another year.

## Systematic review

### Methods

This systematic review was registered on the PROSPERO database (CRD42023400677) prior to the commencement of the search. The Medline and Scopus databases were searched for the terms “sigmoid neovagina”, ‘‘sigmoid vaginoplasty’’ and “prolapse”. Article references were then individually searched to find additional reports. We did not apply any restriction regarding the year of publication or language; however, only results in English were retrieved. The main inclusion criteria were clinical publications of patients with a sigmoid neovagina who developed postoperative prolapse containing the description of the surgery performed to correct the prolapse and the time to recurrence when applicable. We excluded publications concerning male-to-female transgender patients and patients with neovagina created with other methods.

### Results

A total of 57 articles were identified in our database search and review of the references. Of these, 43 were excluded (25 duplicates, three irrelevant to the topic, five referred to male-to-female patients, six for neovagina obtained with other procedures, and four for reporting other complications in place of the prolapse). Fourteen studies, comprised of case reports and case series, were included in our final review, comprising 17 cases of sigmoid neovagina prolapse (SNP); ours was the eighteenth (Fig. 1).

Overall, the median age at which the neovagina construction was carried out was 22 years, and for the prolapse presentation was 45 (32–56) years, ranging from 17 to 73 years (Table 1). POP-Q classification system was retrieved when reported and calculated by the available information, when possible, in 17 cases. Most cases presented with a stage 4 (11/17) prolapse, and the rest were stage 3 (6/17) (Table 1). The total vaginal length was reported only in nine reports, and the median (IQR) length was 9 (7.25–11.75) cm, with one case having a 20-cm TVL (Table 1). The median (IQR) interval time from the surgical creation of the neovagina to the prolapse was 25 (6–32.5) years, with one patient experiencing the prolapse on the very same day as the vaginoplasty (Table 1). Two patients waited more than 10 years before receiving surgical repair of their prolapse, while overall 2 years was the median time from prolapse to correction (Table 1). Recurrence either by previous surgical correction attempts or by the authors' surgery was reported in five cases, which corresponds to a 29% recurrence rate. Some patients required more attempts for definitive correction, with one case requiring a total of five surgeries for complete resolution, and two cases requiring three surgeries, for a total of 11 recurrences in 27 surgeries, which represents a 41% rate (Table 2). When we looked at the types of surgery, we defined them as “definitive” if they were the last surgery without evidence of recurrence in the follow-up reported by the authors. Vaginal resection of the redundant sigmoid, comprising Altemeier’s procedure, either alone or in conjunction with other techniques was the most definitive, but it was also associated with recurrences in three cases. Laparoscopic sacropexy was the second most definitive surgery, with no recurrence reported. The median (IQR) follow-up time from the definitive surgery was 9.5 (6–17) months (Table 2).

**Table 1:** Results of the systematic review of studies on sigmoid neovagina prolapse: timing of the prolapse and staging

Authors (year)	Cases total	N°	Age at vaginoplasty	Age at prolapse	Time from neovagina to prolapse (years)		POP-Q stage	Total vaginal length (centimeters)	Time from symptoms to correction (years)
Novak (1978) [2]	2	1	/	/	/		3	/	/
Novak (1978) [2]	2	2	/	/	/		3	/	/
Freundt (1994) [3]	2	1	/	/	4		4	/	0
Freundt (1994) [3]	2	2	/	/	0		4	/	/
Matsui (1999) [4]	1		24	57	33		/	/	/
Yokomizo (2002) [5]	1		22	25	3		4	/	11
Yokomizo (2002) [5]	1		17	23	6		3	20	15
Tanaka (2003) [6]	1		22	61	40		3	10	2
Kondo (2012) [7]	1		34	40	6		4	/	0
Zhu (2013) [8]	1		22	47	25		4	8	1
Swenson (2014) [9]	1		17	17	0.3		4	11	0
Henningher (2015) [10]	1		17	39	22		3	7	2
Popov (2016) [11]	1		21	72	51		4	7	0
Hao (2017) [12]	1		18	43	25		4	16	5
Fechter (2020) [13]	1		15	48	32		4	8	0
Jiao (2021) [14]	1		22	30	8		4	/	2
Yadav (2021) [15]	1		27	54	27		4	7	5
Drusany (2023)	1		22	73	51		3	12	2
Total			18						
Median (IQR)			1	22 (17.5–22)	45 (32.25–56.25)	25 (6 –32.5)	4 (3–4)	9 (7.25–11.75)	2 (0–4.25)

**Table 2:** Results of the systematic review of studies on sigmoid neovagina prolapse: surgeries and recurrences

Authors (year)	Total cases	N°	First attempt to correction	Definitive correction	Recurrences per each patient	Total number of surgeries for resolution	Follow-up (months)
Novak (1978) [2]	2	1	Vag resection of redundant sigmoid	Vag resection of redundant sigmoid	0	1	/
Novak (1978) [2]	2	1	Vag resection of redundant sigmoid	Vag resection of redundant sigmoid	0	1	/
Freundt (1994) [3]	2	1	Abd suspension to Cooper ligament	Abd suspension to Cooper ligament	0	1	/
Freundt (1994) [3]	2	1	Vag resection of redundant sigmoid	Vag resection of redundant sigmoid	2	3	/
Matsui (1999) [4]	1	1	Abd sacrocolpopexy with mesh	Abd sacrocolpopexy with mesh	0	1	/
Yokomizo (2002) [5]	2	1	Abd suspension using strips of the external oblique aponeurosis + vag resection of redundant mucosa	/	1	/	/
Yokomizo (2002) [5]	2	1	Abd resection of the neovagina + reconstruction of neovagina with vulvoperineal fasciocutaneous flaps	Abd resection of the neovagina + reconstruction of neovagina with vulvoperineal fasciocutaneous flaps	0	1	13
Tanaka (2003) [6]	1	1	Altemeier procedure	Altemeier procedure	0	1	/
Kondo (2012) [7]	1	1	Abd sacropexy w mesh	LPS sacropexy	2	3	6
Zhu (2013) [8]	1	1	Vag bilateral iliococcygeus fascia fixation	Vag bilateral iliococcygeus fascia fixation	0	1	24
Swenson (2014) [9]	1	1	Abd colpopexy to round and utero-ovarian ligaments	Vag sacrospinous suspension	4	5	14
Henningher (2015) [10]	1	1	LPS sacropexy	LPS sacropexy	0	1	18
Popov (2016) [11]	1	1	LPS sacropexy	LPS sacropexy	0	1	6
Hao (2017) [12]	1	1	Vag sacrospinus suspesion + resection redundant mucosa	Vag sacrospinus suspesion + resection redundant mucosa	0	1	6
Fechter (2020) [13]	1	1	Vag bilateral iliococcygeus suspension	Altemeier Procedure	2	3	"several"
Jiao (2021) [14]	1	1	LPS sacropexy	LPS sacropexy	0	1	6
Yadav (2021) [15]	1	1	LPS sacropexy	LPS sacropexy	0	1	36
Drusany (2023)	1	1	Altemeier procedure	Altemeier procedure	0	1	6
Total			/	/	11	27	/
Median (IQR)					0 (0–1)	1 (1–1.5)	9.5 (6–17)

Vag: vaginal; Abd: abdominali; LPS: laparoscpic

## Discussion

### Vaginoplasty

The construction of a neovagina may be necessary for various reasons such as radical surgery, male-to-female transition, or vaginal aplasia as in the case described.

Vaginal aplasia is a very rare condition, in most cases due to Mayer–Rokitansky–Küster–Hauser syndrome (MRKHS), which has an incidence of 1 in 4500–5000 females, but it could also present as an isolated total or segmental vaginal atresia, whose incidence is difficult to estimate [16, 17].

According to the latest guidelines from the American College of Obstetrics and Gynecology (ACOG), the realization of a neovagina using non-surgical methods is the first option and is to be preferred to surgical treatment because it is burdened with a lower rate of complications and because it is more cost-effective [17]. Non-surgical methods are mainly represented by vaginal elongation through self-dilatation. Several surgical treatments have been historically proposed, but most of them require ongoing postoperative dilation or vaginal intercourse in order to avoid stenosis or shrinkage of the vagina, which makes the use of surgery questionable [16]. Bowel vaginoplasty, which uses segments of the small and large intestine to create a neovagina, has the advantages of natural lubrication, a low rate of shrinkage and adequate vaginal length. The sigmoid colon is the segment most often used because of its proximity to the vagina opening and the mobility of its vasculature pedicle. Nonetheless, this surgery is still burdened by complications, one of which is prolapse.

### Incidence and etiopathology

The incidence of sigmoid neovagina prolapse (SNP) is difficult to ascertain given its rarity. In addition, some authors distinguish between full-thickness and mucosal prolapse without defining what distinguishes one from the other [12, 18–20]. Novak et al. were the first to describe the benefits of using the sigmoid colon for artificial vaginas, back in 1978, and in their case series of 95 patients over a 20-year period, only two had a prolapse [2]. According to a systematic review of the case series published from 1992 to 2011, accounting for a total of 560 patients, the cumulative incidence was 2.3% [7]. We are sure that this number underestimates the real incidence, because all these studies have a short mean follow-up period that reaches a maximum of 6 years, such as in the series by Imparato et al. [18]. In contrast, our systematic review shows that the median time from the neovagina to the occurrence of the prolapse is 25 years and, as in our case, it can manifest after more than 50 years (Table 1). Indeed, a recent survey demonstrated that up to 10% of women with MRKH syndrome develop a prolapse, suggesting also that MRKH itself is a risk factor for developing a prolapse [21]. That is partially explained by the fact that the apical and lateral anatomical supports do not develop in these patients [22]. However, the reason why only some patients develop a prolapse is still unknown. The etiopathology is probably multifactorial since it has been attributed to several factors. Kondo has suggested that sexual activity can result in the lengthening of the neovagina. Although plausible, only two cases in the literature reported a total vaginal length that could indicate that (20 cm and 16 cm), while according to our review, the median TVL was 9 cm which is comparable to the average 9.6 cm reported by a large epidemiologic study [5, 7, 12]. Most authors looked at SNP cases as normal vaginal prolapses, and consider the lack of preventive fixation to the first support level structures to be the root of the problem [12, 23]. Tanaka, on the contrary, theorizes that the prolapse may be caused by hyperplasia of the colonic mucosa based on the histopathology report of the resected sigmoid [6]. No other authors reported similar histological findings, and in our case, there were no signs of hyperplasia but only non-specific inflammatory changes that could be explained by friction between the patient’s clothes and the prolapsed mucosa [24]. It is our opinion that the loosening of mucosal support structure as well as an inadequate suspension at the time of the first surgery play an important role.

### Treatment and recurrences

Being a rare condition and—as already stated—little reported, there is no evidence-based recommendation on the best treatment. According to two Cochrane reviews, sacral colpopexy is superior to sacrospinous fixation in patients with a normal vagina in terms of recurrences [25, 26]. As previously mentioned, laparoscopic sacral colpopexy was found to be the definitive treatment—without recurrences—in the majority of cases analyzed in our review. However, this procedure may not always be feasible. Extensive adhesions could make dissecting the walls of the vagina difficult. A vascular stalk that is too high or the position of the sigmoid mesentery may not allow for proper mesh placement [9]. Additionally, due to the thinness of the sigmoid mucosa, the risk of erosion is greater [10, 11].

Given the difficulties encountered in our case, we had to reevaluate our approach and think outside the box. After evaluating the clinical case with a gastrointestinal surgeon, we decided to tackle the prolapse as if it were rectal. The latest Cochrane review on rectal prolapse is not definitive in saying what the best treatment is [27]. The Altemeier approach is widely used, even though it is usually reserved for more fragile patients [28]. This seems to be the third case of SNP treated with an Altemeier procedure. As stated in our results section, we have grouped the Altemeier procedure with other surgeries defined simply as resection of the redundant sigmoid, and according to the data from our review this seems to be the second procedure with the lowest number of recurrences, although a major limitation is that seven studies did not give enough details on the follow-up. Nonetheless, given the satisfactory result obtained, we feel confident in recommending this approach if laparoscopic sacral colpopexy cannot be performed.

## Conclusions

In conclusion, the incidence of sigmoid neovagina prolapse (SNP) is difficult to determine, but it is believed to be underreported due to short follow-up periods in previous case series. The exact etiology of SNP is still unknown and thought to be multifactorial, including factors such as lack of preventive fixation, loosening of mucosal support structures, and sexual activity.

Current treatments for SNP, including sacral colpopexy and sacrospinous fixation, have varying levels of success and may not always be feasible due to anatomical constraints. Our systematic review shows that laparoscopic sacral colpopexy was found to be the definitive treatment without recurrences in most cases analyzed. However, in some challenging cases, the Altemeier approach was used successfully. Further research and larger case series are necessary to establish evidence-based recommendations for the management of SNP.

## Data Availability

All data are included in the medical record of the patient. Clinical data are available from the corresponding author but only on reasonable request.
